# Effects of two different instructional formats on scores and reliability of a script concordance test

**DOI:** 10.1007/s40037-012-0017-0

**Published:** 2012-08-21

**Authors:** W. E. Sjoukje van den Broek, Marianne V. van Asperen, Eugène Custers, Gerlof D. Valk, Olle Th. J. ten Cate

**Affiliations:** 1 School of Medical Sciences, University Medical Center Utrecht, Utrecht, the Netherlands; 2 Center for Research and Development of Education, University Medical Center Utrecht, Utrecht, the Netherlands; 3 Mentrum Institute for Mental Health, Amsterdam, the Netherlands; 4 Department of Internal Medicine, University Medical Center Utrecht, Utrecht, the Netherlands; 5 Center for Research and Development of Education, University Medical Center Utrecht, Room HB 4.05, PO Box 85500, 3508 GA Utrecht, the Netherlands

**Keywords:** Script concordance test, Clinical reasoning, Test instruction, Test reliability

## Abstract

The script concordance test (SCT) is designed to assess clinical reasoning by adapting the likelihood of a case diagnosis, based on provided new information. In the standard instructions students are asked to exclude alternative diagnoses they have in mind when answering the questions, but it might be more authentic to include these. Fifty-nine final-year medical students completed an SCT. Twenty-nine were asked to take their differential diagnosis into account (adapted instructions). Thirty students were asked not to consider other diagnoses (standard instructions). All participants were asked to indicate for each question whether they were confused answering it with the given instructions (‘confusion indication’). Mean score of the test with the adapted instructions was 81.5 (SD 3.8) and of the test with the standard instructions 82.9 (SD 5.0) (*p* = 0.220). Cronbach’s alpha was 0.39 for the adapted instructions and 0.66 for the standard instructions. The mean number of confusion indications was 4.2 (SD 4.4) per student for the adapted instructions and 16.7 (SD 28.5) for the standard instructions (*p* = 0.139). Our attempt to improve SCTs reliability by modifying the instructions did not lead to a higher alpha; therefore we do not recommend this change in the instructional format.

## Introduction

Clinical reasoning is considered a central component of a physician’s medical expertise and there is broad consensus that it should be taught and tested in medical curricula [1, 2]. One test format that has received considerable attention in the past decade of medical education literature is the script concordance test (SCT) [3]. This test is based on clinical knowledge organised in illness scripts: a physician compares the presenting patient to fairly similar cases he or she has encountered in the past and uses these experiences to efficiently make judgments regarding the present case [4].

The SCT is a relatively recent innovative method to assess clinical reasoning skills [5]. Evidence for its validity is available, but it still has to be further confirmed [3, 6, 7]. The SCT uses a closed-answer format to assess clinical reasoning and stimulates the test taker to think of realistic clinical scenarios in which candidates are asked to interpret data to make clinical decisions [4, 5]. In an SCTs clinical scenario a patient’s history, the results of physical examination and, occasionally, the results of diagnostic tests are provided. In addition, a diagnostic hypothesis is proposed. Next, the test taker is presented with a new clinical finding that could alter the likelihood of this hypothesis, i.e. making it potentially more or less likely, or not affect it. The response (answer) is made on a five-point Likert scale, ranging from ‘makes the diagnostic hypothesis much more likely’ through ‘does not change the probability of the hypothesis’ to ‘makes the diagnostic hypothesis much less likely’.

A unique feature of the SCT is that there is no predetermined ‘right’ or ‘wrong’ answer to each question, but that the examinee’s response is compared with the average response of a panel of experts, which is used to construct the scoring key. The score assigned to every response alternative corresponds with the proportion of experts choosing this alternative. This scoring system is designed to measure the difference in existing scripts between examinees and this panel [4]. In other words, the scoring is weighed by the degree of agreement between the experts [8].

The SCT has been applied in several target groups [6, 9–11], among which pre-clinical medical students [12, 13]. At the University Medical Center Utrecht, the SCT was applied among second-year medical students, as a final test of a course in case-based clinical reasoning in 2009–2010. Students’ evaluations of the SCT revealed that they appeared to be confused about the instructional format. The issue at hand is that new information in a clinical case may not directly influence the probability of a focal diagnostic hypothesis—for instance, it may not bear upon this hypothesis—but may have an indirect influence by making an alternative diagnostic hypothesis more or less likely, with a concomitant change in likelihood of the focal hypothesis.

The standard instructions for the SCT, which we used for the test in 2009–2010, prescribe that candidates should not consider alternative hypotheses when assessing the change in probability of the focal hypothesis as a consequence of the new information [14]. Our evaluations indicated that second-year students have difficulty working with these instructions. An example can explain this: ‘A 72-year-old lady, known with rheumatoid arthritis, presents at the general practice surgery with a swollen knee. Given only this information, the suggested hypothesis is: inflammation of the joint.’ The new information is: ‘she fell off her bicycle 2 h ago’. Question: how does this new finding affect the likelihood of the suggested hypothesis? Respondents must choose one alternative: ‘makes it less likely’, ‘makes no difference’ or ‘makes it more likely’. With the standard instructions for the SCT, students should answer that falling off the bicycle does not affect the likelihood of an inflammation. However, it is imaginable that students find it difficult and unnatural to choose this answer, as in reality they would now much more readily think of a trauma, and therefore find an inflammation less likely. To summarize: the likelihood of a diagnostic hypothesis, as perceived by a respondent, may not only be affected by a causal relation to the signs and symptoms in the case, but also by its ranking among alternative hypotheses. The instructional format of the SCT could alternatively be phrased as ‘keep other diagnoses you find plausible in mind when evaluating the changes in likelihood of the proposed diagnosis’. We would expect that students would find this more natural and would be less confused. This could subsequently be reflected in a higher reliability, as it could reduce error variance caused by confusion and also yield a higher validity as it resembles the authentic setting better, possibly resulting in higher scores.

Thus, in our study we investigate whether the adapted instruction to consider alternative diagnoses when answering the questions in an SCT results in higher scores and increased reliability compared with the standard instruction not to take other diagnoses into account when assessing changes in likelihood.

## Methods

### Participants

To examine the effects of instructions on scores and reliability of the SCT, a panel of 59 final-year medical students (from a final-year course) and 18 experts (general practitioners), completed an SCT. We invited final-year medical students because we assumed they could answer the SCT questions without specially preparing for the test, whereas junior students would not be able to do this. All students and experts participated voluntarily and data were processed anonymously.

### Materials

Two versions of the test were prepared, each of which contained 10 clinical vignettes, each accompanied by multiple diagnostic hypotheses in subsequent questions. The 10 vignettes represent 10 general medical complaints students are familiar with (for example, headache and back pain). The test contained 100 questions, distributed over the 10 vignettes, varying from 7 to 13 questions for each vignette. The questions for each separate vignette could be answered independently (i.e., the questions are not chained or ordered). In our SCT, we used a three-point Likert scale: less likely—no difference—more likely, rather than the original five-point scale. From experience we know that junior students have difficulty dealing with relatively subtle distinctions, such as the one between ‘slightly more likely’ and ‘much more likely.’

The different versions of the SCT test differed only in the instructions given to the test takers (standard instructions vs. adapted instructions, see below).

### Procedure

Students as well as experts were randomized into two groups, by separating even and odd numbers of the alphabetical subject list (students) or by lot (experts). The students were all unfamiliar with the SCT test and received oral instructions from the same supervisor, though in separate sessions. The instructions also appeared in written form on the first page of the respective test versions. Students were not informed that the experimental manipulation concerned two different instructional formats. They were asked to complete the test within 1 h.

The members of the two expert groups used a digital questionnaire programme (Evasys^®^) to complete the test. Like the students, they also received either the standard or the adapted instructions but only on paper. The results of the two respective expert panels were used to determine the scoring key for each version of the test. As the average response of an expert panel determines the standard for the test questions in the SCT, two different expert panels were used for the two test versions.

### Instructions

The following instructions were given: ‘This test contains 100 questions in the following format: (1) a short case scenario and (2) a diagnostic hypothesis. Next, you will receive (3) new, additional data. Then state whether the diagnostic hypothesis becomes more likely, less likely, or that the likelihood of the hypothesis is not influenced by this additional data’.

For the groups with the adapted instructions (29 students and 9 experts) these instructions ended with: ‘Take into account your differential diagnosis when answering the questions’. For the group with the standard instructions (30 student and 9 experts) the instructions ended with: ‘Do not take other diagnoses into account when answering the questions’. An example was also given. This example, with the two different instructions, is shown in Table 1.

The adapted instruction to take into account the differential diagnosis or the standard instruction to consider other diagnoses as excluded was repeated in each separate question as a reminder.

All participants were asked to indicate for each question (in a checkbox) whether they felt confused and therefore had difficulty answering the question with the given instructions.

The expert panel had the option to write comments in a textbox at the end of the digital test. The opinion of the students was asked in an open discussion immediately after finishing the test.

### Data handling and data analysis

Data were collected for each student group separately. Individual scores and mean scores were calculated. Mean scores were compared using an independent Student’s *t* test. The distribution of the scores justified the use of a parametric test.

Cronbach’s alpha was calculated for both groups of final-year students individually. The number of times the ‘no influence’ option was chosen and the number of questions for which students ticked the confusion checkbox were separately registered for each group. The mean numbers of confusion indications for both tests were compared using a Mann–Whitney *U* test.

### Ethical considerations

At the time this study was performed, ethical approval was not required in the Netherlands for medical education studies. All students and experts were informed about the study and asked to participate voluntarily. There were no risks for the participants in this survey. Individual results of the students’ tests were collected anonymously and could not affect students’ progress in any way. This was communicated orally to the participants.

## Results

All students were able to complete the test within approximately 1 h.

For some questions all three answer options were chosen by at least one member of the expert panel. Because the scoring for each question is weighed by the degree of agreement between the experts [8], participants could receive some points for every answer option they chose for these questions. As a result, the lowest obtainable score for the tests was not zero points but 5.76 for the test with the adapted instructions and 5.92 for the test with the standard instructions. The maximum score was 100 points.

The results for both groups of students are shown in Table 2.

**Table 1:** Instructional example of the SCT questions with the two different formats as provided to the respective groups in this study

Case scenario
**A mother of a 15-month-old boy visits your consulting hour because her son is crying all the time and has been agitated for two days now**
Hypothesis: **Constipation**
Additional data: **He is constantly tugging at his right ear**
With this new additional data the hypothesis:
(Adapted instructions: ‘Take into account the differential diagnosis’)
(Standard instructions: ‘Consider other diagnoses as excluded’)
**• Becomes more likely**
**• Is not influenced**
**• Becomes less likely**
For the **groups with the adapted instructions** the following was added: *You may consider an inflammation of the ear as a likely diagnosis. As a consequence, the hypothesis ‘constipation’ will move to a lower position in your differential diagnosis. ‘Becomes less likely’ could therefore be your answer to this question*
For the **groups with the standard instructions** the following was added: *You may consider an inflammation of the ear as a likely diagnosis. However, do not take this diagnosis into account when answering the question. Constipation in itself does not become more or less likely by the tugging at an ear. ‘Is not influenced’ could therefore be your answer to this question*

**Table 2:** Results for the SCT (for the students) with the two different instructions

	Test with adapted instructions (include differential diagnosis when answering the questions)	Test with standard instructions (exclude other diagnoses when answering the questions)	*p* value
	*N* = 29; 100 questions per test	*N* = 30; 100 questions per test	
Mean score (SD)	81.50 (3.80)	82.94 (5.01)	*p* = 0.220 (independent Student’s *t* test)
Range: 5.76–100 test with adapted instructions, 5.92–100 test with standard instructions	73.18–89.10	70.12–92.08	
Cronbach’s alpha	0.388	0.655	
Mean number of confusion indications (SD)	4.17 (4.42)	16.70 (28.47)	Mann–Whitney test Z_*U*_ = −1,481 *p* = 0.139
Answer option ‘no influence’ chosen (% of all responses)	23.3 %	40.2 %	*p* < 0.001 (independent Student’s *t* test)

Students in the standard instructions group and in the adapted instructions group showed similar performance: 82.94 (±5.01) points and 81.50 (±3.80) points, respectively (*p* = 0.220).

The Cronbach’s alpha reliability was low for both tests: 0.388 for the test with the instruction to include other diagnoses when answering the questions (adapted instructions), and 0.655 for the test with the instruction to exclude other diagnoses than the presented hypothesis when answering the questions (standard instructions).

The mean number of questions with a confusion indication was 4.17 (SD 4.42) per student for the group with the adapted instructions, and 16.70 (SD 28.47) for the group with the standard instructions (to exclude other diagnoses when answering the questions). As three students in the standard instructions group indicated that they experienced confusion for almost all the questions, we believed a *t* test of group means to be potentially misleading and performed a Mann–Whitney *U* test, which rank orders students according to the number of questions they were confused about. This test revealed no significant difference between the groups (Z_*U*_ = −1,481; *p* = 0.139).

Students who completed the SCT version with the adapted instructions (i.e., include other diagnoses in assessing likelihood changes) were considerably less inclined to chose the answer option ‘the hypothesis is not influenced by the new additional data’ (a statistically significant difference of 23.3 % of questions for the group with the adapted instructions versus 40.2 % for the group with the standard instructions).

During the debriefing, students who received the standard instructions noted that it felt unnatural for them to exclude alternative diagnostic hypotheses, as this does not reflect the way doctors think. Also four (out of nine) experts who received these instructions noted the same in the comment box in the digital test.

## Conclusion and discussion

In the standard instructions for an SCT students are asked to exclude alternative diagnoses they have in mind when answering the questions. However, the likelihood of a provided diagnostic hypothesis in an SCT question may not only be affected by the causal relation to the signs and symptoms in the case, but also by its ranking among alternative hypotheses. Therefore, we designed an adapted instruction—to take other diagnoses into account when evaluating the changed likelihood of a provided hypothesis. This difference in instructional format does not affect the mean scores of the SCT. Though not significant, there is a tendency for students to be less confused when answering questions in the adapted instructions, compared with the standard instructions. Reliability in terms of internal consistency, of the adapted form, however, appears to be lower in our study (Cronbach’s alpha 0.388 vs. 0.655). Finally, the option ‘no influence on likelihood of focal hypothesis’ is less appealing in the adapted instructions version of the SCT.

In line with our expectations, students and members of the expert panel who received the standard instructions commented that it felt unnatural for them to exclude a differential diagnosis while answering the questions.

Contrary to our expectations, the instruction to include a differential diagnosis yielded a considerably lower alpha (0.39) than the instruction to exclude other diagnoses (0.66). We had assumed that if students are allowed to consider a case-triggered differential diagnosis while answering diagnostic questions, which resembles reality, this would improve reliability. We do not find evidence for this assumption.

It is not clear what causes the large difference between the alphas of the tests. In fact, the reliability of both test versions can be regarded too low to consider the SCT a valid summative assessment tool, in particular given the fact that it is a rather extensive test which takes participants 1 h to complete [15].

Participants who were instructed to consider a differential diagnosis (adapted instructions) while judging the value of new information, choose the option ‘the likelihood of the diagnosis is not influenced’ considerably less often than the group with the standard instructions (23.3 vs. 40.2 %, see Table 2). In all likelihood, this is a consequence of the adapted instructions. The additional data in most cases influence at least one of the diagnoses students have in mind. This results in a shift in their differential diagnosis. Then, the provided focal hypothesis automatically moves up or down in the list with possible diagnoses, to more or less likely. However, students explained in the open discussion that this answer option had caused some confusion. When the additional data cause only a small shift of the presented hypothesis, students did not know whether to answer ‘becomes more/less likely’ or ‘is not influenced’. This problem may not have occurred if the original five-point Likert scale was used, varying from very unlikely to very likely, as students would then have had more possibilities to indicate the extent of the shift in likelihood of the focal diagnosis.

In addition, participants may easily weigh one hypothesis at a time against the new information, but may have difficulty in doing this for multiple diagnoses simultaneously.

We do not know which other diagnoses were in the minds of the participants when answering this type of question. It would be interesting to investigate this for both students and expert panel members and this could add to the evaluation of participants’ knowledge.

It should be noted that students’ mean scores on both versions of the SCT were relatively high. This might be a consequence of the scoring format that was used, a three-point scale, rather than the original five-point scale. Given the multiple choice nature of the items, a three-point scale leads to a lower reliability than a five-point scale due to guessing. The same guessing would more easily lead to high scores. In addition, the SCT-scoring procedure awards partial credits to answers that are chosen by a minority of panel members, which may inflate the overall level of the scores.

Another explanation could be that the test was designed for second-year students and taken by final-year students. This could have led to a restriction in the range, as the test was not very difficult for this population (Table 2). The more homogeneous, high scores of final-year students could have decreased the discriminating power of the test in both conditions, consequently lowering alpha. In addition, the two 9-member panels could be considered relatively small, negatively affecting Cronbach’s alpha [15]. A test with more questions (e.g. 200 questions) would probably have resulted in satisfactory reliability in the control condition, estimated with the Spearman-Brown formula (0.79 for a test with 200 questions).

For further validation it would also be useful to repeat the study with different target groups, in particular second-year students, the population that caused our original concern. And, as mentioned before, it would be interesting to investigate which differential diagnosis participants have in mind when answering the questions, for both students and expert panel members [16, 17]. Finally, Lubarsky et al. [3] concluded that evidence supporting the validity of SCT scores with respect to examinees’ thought and response processes is still limited.

In sum, we conclude that the instruction to consider alternative diagnoses when answering SCT questions, though reflecting a higher level of authenticity according to students and experts, does not improve reliability. On the contrary, it appears to negatively affect its reliability. Here is where validity, in the sense of authenticity, seems in conflict with the classic approach to test reliability. One explanation could be that clinical reasoning is not a unitary concept and hence, Cronbach’s alpha, a measure of internal consistency, is not the best way to assess the SCTs reliability. Unlike most other tests used for assessment purposes in medical education, the SCT does not measure knowledge, application, or insight, but purports to measure (clinical) reasoning. A well-known phenomenon in the domain of clinical reasoning is *case specificity*, i.e., low correlations between diagnostic performance in individual cases [18]. Before dismissing the SCT as a less useful tool to assess clinical reasoning, this issue should be further investigated.

## Essentials

- The standard instructions in the SCT, to exclude alternative diagnoses in assessing the impact of new information on the likelihood of a particular diagnosis, could cause confusion among test takers as this does not reflect real-life clinical reasoning.
- The adapted instructions to include a differential diagnosis when answering the questions lead to less experienced confusion but also to a lower test reliability (Cronbach’s alpha).
- Cronbach’s alpha, a measure of internal consistency, might not be the best way to assess SCTs reliability, as clinical reasoning may not be a unitary concept.
